# Novel Use of Natural Language Processing (NLP) to Predict Suicidal Ideation and Psychiatric Symptoms in a Text-Based Mental Health Intervention in Madrid

**DOI:** 10.1155/2016/8708434

**Published:** 2016-09-26

**Authors:** Benjamin L. Cook, Ana M. Progovac, Pei Chen, Brian Mullin, Sherry Hou, Enrique Baca-Garcia

**Affiliations:** ^1^Health Equity Research Laboratory, Cambridge Health Alliance, Department of Psychiatry, Harvard Medical School, 1035 Cambridge Street, Suite 26, Cambridge, MA 02141, USA; ^2^Wired Informatics, 265 Franklin Street, Suite 1702, Boston, MA 02110, USA; ^3^Hospital Universitario Fundación Jiménez Díaz, Avda. Reyes Católicos 2, 28040 Madrid, Spain; ^4^Autonomous University of Madrid, Ciudad Universitaria de Cantoblanco, 28049 Madrid, Spain

## Abstract

Natural language processing (NLP) and machine learning were used to predict suicidal ideation and heightened psychiatric symptoms among adults recently discharged from psychiatric inpatient or emergency room settings in Madrid, Spain. Participants responded to structured mental and physical health instruments at multiple follow-up points. Outcome variables of interest were suicidal ideation and psychiatric symptoms (GHQ-12). Predictor variables included structured items (e.g., relating to sleep and well-being) and responses to one unstructured question, “how do you feel today?” We compared NLP-based models using the unstructured question with logistic regression prediction models using structured data. The PPV, sensitivity, and specificity for NLP-based models of suicidal ideation were 0.61, 0.56, and 0.57, respectively, compared to 0.73, 0.76, and 0.62 of structured data-based models. The PPV, sensitivity, and specificity for NLP-based models of heightened psychiatric symptoms (GHQ-12 ≥ 4) were 0.56, 0.59, and 0.60, respectively, compared to 0.79, 0.79, and 0.85 in structured models. NLP-based models were able to generate relatively high predictive values based solely on responses to a simple general mood question. These models have promise for rapidly identifying persons at risk of suicide or psychological distress and could provide a low-cost screening alternative in settings where lengthy structured item surveys are not feasible.

## 1. Introduction

Suicide is the 13th leading cause of death globally, accounting for 5-6% of all deaths [1]. The risk of completed suicide varies worldwide by various sociodemographic characteristics, with young adults, teens, and males facing the highest risks of suicide completion [2–7]. Despite the fact that females are significantly more likely than males to make a suicide plan, male suicide attempts are more likely to result in death, and the male-female disparity in completed suicides is most pronounced in higher-income countries [8]. Unlike the risks of many health conditions, which are lower in more developed nations, suicide rates are higher in areas with advanced health systems [9]. Concerns about the impact of suicide have become particularly salient in recent years as citizens in countries worldwide have faced the brunt of the financial crisis in 2008. Spain, which was hit especially hard by this economic downturn compared to other European countries, experienced bimodal jumps in suicide in line with the double-dip recession experienced there and elsewhere in Europe [5]. The long-term detrimental impacts of suicide and suicidal ideation on individuals, families, and communities are difficult to estimate and likely vary by socioeconomic status worldwide.

In its first Mental Health Action Plan report in 2014 [8], the World Health Organization (WHO) recognized that better data from registries, hospital records, and surveys are key components in reducing suicide attempts and mortality. Innovative and economically feasible strategies to collect and interpret data for suicide prevention purposes are critical tools in suicide prevention efforts. Computational methods such as natural language processing (NLP) combined with machine learning prediction techniques that utilize existing data from Electronic Health Records (EHRs) and other registries have the potential to help meet the goal of identifying and intervening with patients with a high likelihood of a suicide attempt, particularly because they may provide a lower-cost alternative to other costly data collection methods [10].

NLP-based prediction using unstructured clinician notes is emerging as a useful tool in improving identification of certain health conditions [11] and treatment resistant mental health problems [12]. This prediction method has also shown preliminary success in predicting adverse health outcomes [13, 14] such as postoperative complications (where it outperforms traditional patient safety indicators [13]). In the mental health field, an actuarial risk algorithm incorporating EHR data was used to predict, with a reasonable amount of certainty, likelihood of suicide 12 months after inpatient psychiatric treatment. Researchers used the algorithm to identify 52.9% of the suicides occurring after inpatient treatment among US soldiers receiving care in the VA setting [15]. EHR data has been successfully used to predict depression diagnosis as much as one year before the diagnosis occurs and to predict differences in how severe this depression will be upon diagnosis [16]. NLP-based prediction technologies have also been used for a range of other biomedical applications such as notifiable diseases [17–19], rapidly identifying slow-healing wounds [20], adverse events [21, 22], medications [23, 24], and detecting comorbidities [25, 26].

We add to this line of research by applying an NLP/machine learning analytical strategy to analyze short message service (SMS, or text messaging) occurring outside of a clinical setting. This work represents the nascent stages of developing and employing a predictive algorithm in a free-text platform (i.e., physician notes in EHRs, texts, and social media) in order to predict suicidal ideation and heightened psychiatric symptoms. Real-time alerts generated from SMS text have the potential to inform timely clinical interventions to prevent suicide and heightened psychological distress.

## 2. Methods

### 2.1. Study Sample

Adults discharged after self-harm from emergency services or after a short hospitalization in a hospital system in Madrid, Spain, were recruited to participate in the intervention over a 12-month period. Inclusion criteria were as follows: male or female, aged 18 or older, surviving a suicide attempt, discharged from emergency department (ED) or psychiatric units (PU), hospitalized for less than 7 days, giving consent, and able to be contacted by phone. The exclusion criteria were refusing to participate and being underage, incarcerated, under guardianship, without a mobile phone, enrolled in other trials, and in emergency situations where patient's state of health made it difficult to obtain written consent. Participation in the study was offered to all suicidal adults referred to the psychiatric ED meeting the abovementioned criteria. Adults who attempted suicide were admitted to the general ED and evaluated by an ED psychiatrist who decided patients' discharge or hospitalization. Patients were enrolled after this evaluation.

### 2.2. Description of Texting Intervention

The intervention was comprised of therapeutic reminders delivered by SMS messages that were sent out two days, seven days, 15 days, and monthly after hospital discharge. Each text message also provided a link to a mobile application that contained a questionnaire eliciting responses related to the patients' sources of help, evidence-based self-help strategies, and structured interview questions related to suicidal ideation, psychiatric symptoms, and satisfaction with care. Participants were also asked one unstructured, open-ended question related to their current mental state, “how are you feeling today?,” and were encouraged to report on their progress since the hospitalization. Participants were able to enter responses to questionnaires up to once per day and were instructed to answer as often as they wished.

### 2.3. Outcome Variables of Interest

The first outcome of interest was suicidal ideation measured by the question “Ha sentido que no tenía ganas de vivir?,” translated in English as “Have you felt that you do not have the will to live?” Six possible responses were nunca (never), de vez en cuando (sometimes), menos de la mitad del tiempo (less than half the time), más de la mitad del tiempo (more than half the time), la mayor parte del tiempo (most of the time), and todo el tiempo (all of the time). This variable was dichotomized as suicidal ideation,* yes* (endorsed suicidal ideation at any point in the study), or* no* (never endorsed suicidal ideation).

The second outcome of interest is heightened psychiatric symptoms as measured by the General Health Questionnaire (GHQ-12), a self-administered screening questionnaire for detecting overall psychological wellbeing and nonpsychotic psychiatric problems [27]. As a severity index for psychological morbidity with unidimensional properties [28], the GHQ-12 has been shown to have high validity and reliability, and its external and structure validity have also been validated in the Spanish general population [29]. Participants responded to items such as “Have you been able to enjoy your normal day to day activities?” and “Have you been feeling unhappy or depressed?” by providing answers such as “more than usual” or “much less than usual” on a 0–3 Likert scale. Total scores range from 0 to 12. Cutoffs in the general populations when using the GHQ-12 as a screening instrument are generally low (2 and above or 3 and above), but for this sample of patients with recent hospitalization for a self-harm, we considered a score of 4 or above to be a more appropriate cutoff point.

### 2.4. Other Covariates Taken from the Mobile Application

Participants also used the cell phone application to report hours of sleep of the previous night, sleep quality of the previous night, appetite, anger/aggression, treatment adherence to medications, and the WHO-5 screening variable. The WHO-5 is a five-item questionnaire measuring subjective wellbeing derived from longer ratings scales used by the WHO in a multicenter, multicountry study in Europe [30]. Participants' responses to questions were recorded using a sliding bar for each question. The range for the sliding bar varied by measure: sleep quantity (the bar could be slid between 0 and 12 hours), sleep quality (bad, regular, or good), appetite (less, no change, or bigger appetite), conflicts or fights (“never” to “all of the time”), and medication treatment adherence (“never take medications” to “take all medications”). The sliding bar feature allowed for a full range of responses along the continuum of each question. Raw scores on the WHO-5 range from 0 to 25 (0 = absence of wellbeing, 25 = maximum wellbeing) and are multiplied by 4 so that values are on a percentage scale from 0 to 100. At a cutoff of ≤50, this scale signals clinical depression with a sensitivity of 0.86 and specificity of 0.81. It is also considered reliable for determining changes to wellbeing over time [30].

### 2.5. Natural Language Processing- (NLP-) Based Machine Learning Tool for Predicting Suicidal Ideation and Psychiatric Problems Using Free Text

Software developed by Wired Informatics was used to conduct higher level semantic processing of the complete text of respondents' answers to the open-ended question, “How are you feeling today?” This software uses the clinical Text Analysis Knowledge Extract System (cTAKES) [31] to generate NLP-based algorithms. Texts from a random half of the sample were used to train the model. During NLP algorithm development, the “*n*-grams” feature was used, meaning that predictions were based upon a contiguous sequence of* n* words (rather than single words with no linguistic context). A number of settings for* n*-gram size (i.e., number of words included in a contiguous string) were tested in order to identify the greatest positive predictive value (PPV) and to increase the number of true positives (increase specificity), recognizing that there was a tradeoff between higher specificity and lower sensitivity. The* n*-gram setting that maximized the PPV was identified as zero words preceding and three words following a “token” word, equivalent to a “trigram.” We found that, in general, bigrams (two words following a token) and single word settings decreased specificity but increased sensitivity.

*n*-grams were then codified and used as inputs to a machine learning (ML) algorithm to predict patients' probabilities of suicidal ideation or heightened psychiatric symptoms (GHQ-12 ≥ 4). The machine learning program uses data from patients with and without these outcomes to train the model using a LIBLINEAR machine learning protocol, a flexible linear classifier that can be used to classify up to millions of instances and features, to develop predictive use cases [32]. Model performance was then evaluated by testing the model's PPV, sensitivity, and specificity on the positive cases in the remaining half of the data not used in the training process.

### 2.6. Statistical Methods

Baseline characteristics of the overall study sample are described, as well as characteristics based on participants' reported suicidal ideation (any versus none) and GHQ-12 average scores. Distributions of all continuous variables were assessed. For continuous variables, two sample* t*-tests compared sample means between participants ever reporting suicidal ideation and those that never reported suicidal ideation. Significant differences in sample means for continuous skewed variables were assessed using Wilcoxon rank-sum (Mann–Whitney) tests, while differences in binary variables between the two groups were assessed using Pearson chi-squared statistics. Multivariate logistic regression models were estimated on the two dependent variables (suicidality and GHQ-12 ≥ 4), conditional on age, sex, nightly sleep hours, sleep quality, anger, appetite, medication adherence, and the WHO-5 scale.

To calculate sensitivity, specificity, and PPV of structured data predictors, we repeated logistic regression models using half of our sample (the same randomly selected “training” dataset used for unstructured data) and then used the predicted probabilities from this logistic regression to characterize the remaining half of our sample as having suicidal ideation or heightened psychiatric symptoms (GHQ-12 ≥ 4) based on the values of their structured data predictors (covariates). Statistical analyses and logistic regression-based predictions were performed using STATA 14 software.

## 3. Results

### 3.1. Sample Characteristics

Participants (*n* = 1,453) had an average age of 40.5 and were 65% female. On scales using a sliding bar for each question and standardized to the general population from 0 to 100 with mean of 50, patients in this sample reported mean adequacy of sleep of 58.8 (SD 14.7), mean sleep quality of 62.3 (SD 26.1), mean score of appetite of 52.1 (SD 21.4), mean score on medication use adherence of 75.7 (SD 35.7), and mean anger/aggression of 74.1 (SD 22.3). Forty-three percent of participants (*n* = 609) never reported suicidal ideation. The average GHQ-12 reported was 4.44 (SD 4.23). The average WHO-5 rating was 48.5 (SD 23.5).

Participants who did not report suicidal ideation during the study (*n* = 609) were on average younger (*p* < 0.01) and less likely to be female (*p* < 0.01) than those who had reported suicidal ideation at some point during the intervention (see Table 1). Participants who never had suicidal ideation slept an average of 7.32 (SD 1.57) hours per night, compared to 6.86 (SD 1.87) hours per night for their peers with suicidal ideation (*p* < 0.01). Sleep quality was also significantly higher for nonsuicidal participants (72.4, SD 24.1) as compared to their peers experiencing suicidal thoughts (55.0, SD 25.1, *p* < 0.01). Overall, nonsuicidal participants were less likely to experience anger/aggression and were slightly more likely to report increases in appetite (both *p* < 0.01), as well as slightly more likely to report adherence to medications (*p* < 0.01). The average WHO-5 wellbeing scale was significantly lower for those with suicidal ideation (38.3, SD 20.0) than for those free from suicidal ideation during the study period (61.6, SD 21.1, *p* < 0.01). Participants with heightened psychiatric symptoms (GHQ-12 ≥ 4) were similar in the majority of characteristics to those who ever reported suicidal ideation (Table 1).

Assessing the two dependent variables simultaneously, 73% of participants that ever reported suicidal ideation had an average GHQ-12 score ≥ 4, compared to only 29.1% of their nonsuicidal peers (*p* < 0.01, see Appendix). Though these two outcome variables demonstrated a great deal of overlap, there were 229 individuals with average GHQ-12 < 4 but who reported suicidal ideation at some point during the study. Similarly, 178 individuals with GHQ-12 ≥ 4 never reported suicidal ideation, confirming the need to examine both outcomes in order to better identify individuals at risk.

### 3.2. Structured Data Predictors of Suicidal Ideation and Psychiatric Symptoms (GHQ-12 ≥ 4)

#### 3.2.1. Suicidal Ideation

In logistic models predicting suicidality, each additional year of age was associated with a 2% higher odds of suicidal ideation in our sample (*p* < 0.01, see Table 2). An additional hour of nightly sleep was associated with 11% higher odds of suicidal ideation (*p* = 0.037). Rarely being angry, as well as higher scores on the WHO-5 wellbeing scale, was associated with 3% and 4% lower odds of suicidal ideation, respectively (both *p* < 0.01). Gender differences in suicidal ideation seen at baseline, sleep quality, changes in appetite, and medication adherence were no longer significant predictors.

#### 3.2.2. GHQ-12

Though hours of sleep were not significant predictors of high GHQ-12 scores, each additional self-reported point on the sleep quality variable (0–100) was associated with 1% lower odds of high GHQ-12 (*p* = 0.029). Rarely being angry and the WHO-5 wellbeing scale were also associated with lower odds of having high average GHQ-12 (both *p* < 0.01). No other structured predictors were significant predictors of having a high GHQ-12 in logistic models.

### 3.3. Free-Text Predictors of Suicidal Ideation

The top 50 words which predicted suicidal ideation ranged from being associated with a 0.38 probability of suicidal ideation to a 0.18 probability of suicidal ideation (see Table 3(a)). The top 10 words associated with suicidal ideation were conté (I told), monotona (monotony), Equasim (Ritalin), acosado (harassed), trabajamos (we work), raza (race), aseos (restrooms), resfriado (congested/sick), pronuncio (I pronounce), and rechaza (rejects). The top 50 words that predict suicidal ideation are represented using word maps (Figure 2 in English and Figure 3 in Spanish, created using Wordle.com), with larger-sized words proportionately weighted based on the probability that these words occur in texts during which a participant reports suicidal ideation. The NLP software allows for negation, trigrams, thus adding important context to these highly predictive tokens.

### 3.4. Free-Text Predictors of GHQ-12 ≥ 4

The top 50 words which predicted heightened psychiatric symptoms (GHQ-12 ≥  4) ranged from being associated with a 0.44 probability of heightened psychiatric symptoms to a 0.16 probability of heightened psychiatric symptoms (see Table 3(b)). The top 10 words associated with heightened psychiatric symptoms were recorrer (wander), horarios (schedules), comunicar (communicate), iré (will go), parecida (similar), gimnasia (gym), destacando (highlight), ido (going), fumada (smoked), llevaré (will bring) (see Figures 4 and 5 for word maps for the top 50 words in English and Spanish).

### 3.5. Predictive Power of Structured Variables versus Unstructured Text Inputs

**Figure 1:**
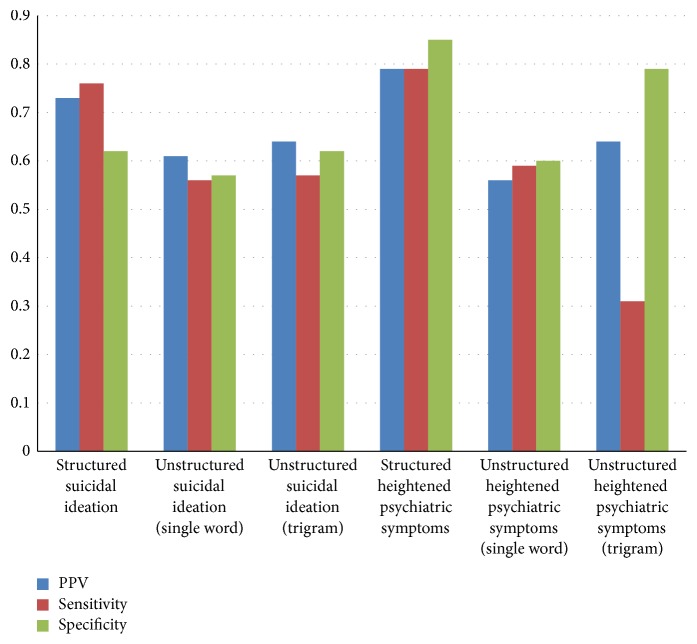
Predictive analytics for structured and unstructured (NLP) models.

**Figure 2:**
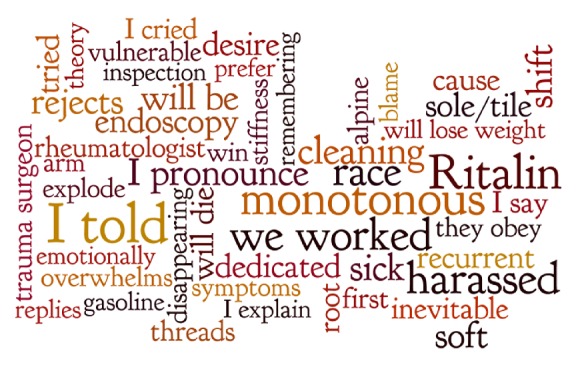
Word map of text-based predictors of suicidal ideation from NLP (English).

**Figure 3:**
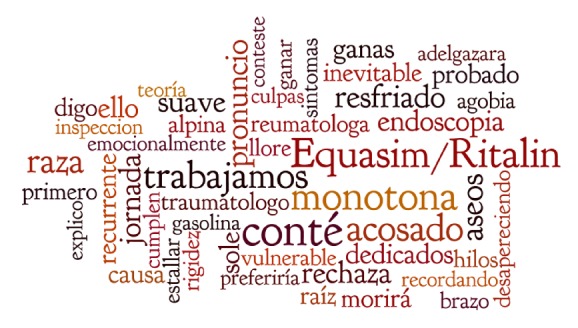
Text-based predictors of suicidal ideation from NLP (Spanish).

**Figure 4:**
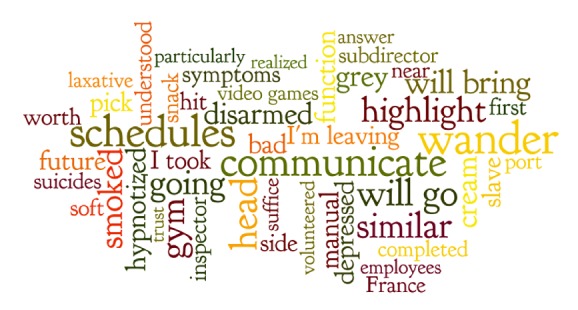
Word map of text-based predictors of GHQ-12 ≥ 4 from NLP (English).

**Figure 5:**
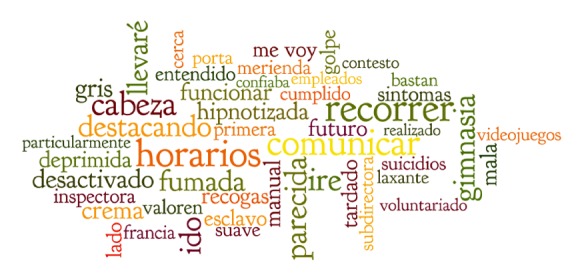
Word map of text-based predictors of GHQ-12 ≥ 4 from NLP (Spanish).

Predictions from the multivariate logistic model using structured covariates for suicidal ideation had a positive predictive value (PPV) of 0.73, sensitivity of 0.76, and specificity of 0.62 (see Figure 1). Using the single token-based NLP/machine learning algorithm to predict suicidal ideation from responses to the open-ended question resulted in a PPV of 0.61, sensitivity of 0.56, and specificity of 0.57. Using the trigram-based algorithm, we found a PPV of 0.64, sensitivity of 0.57, and specificity of 0.62.

For predicting heightened psychiatric symptoms (GHQ-12 ≥ 4), the structured data model had a PPV of 0.79, sensitivity of 0.79, and specificity of 0.85. The single token-based NLP/machine learning algorithm for heightened psychiatric symptoms had a PPV of 0.56, sensitivity of 0.59, and specificity of 0.60. Using the trigram-based algorithm, we found a PPV of 0.64, sensitivity of 0.31, and specificity of 0.79.

## 4. Discussion

### 4.1. Summary of Findings and Contribution to the Literature

Our analysis found that a NLP-based machine learning model using only open-ended texts from patients had a reasonably high predictive value for suicidal ideation and heightened psychiatric symptoms. The structured data performed better, with higher PPV and better sensitivity and specificity, but with the tradeoff that these structured data require significantly more time from the respondent. These findings suggest that even data obtained from free-text responses to general questions about patients' mental state could be used to effectively predict suicidal ideation using computational analytics such as NLP.

This type of NLP-based modeling has applicability to other social media data that are publicly available and shared (Facebook, Twitter, and Instagram). In clinical settings, patients routinely share text-based information with physicians over secure messaging platforms, and tools could be used in such settings to help identify potentially suicidal or distressed patients and to prevent adverse outcomes.

The preliminary evidence from this project demonstrates that NLP-based modeling has the potential to be an important tool for preventing suicide among young adults that frequently use text or social media. The technology could be extended to generate risk flags for patient messages sent to physicians, who could then intervene for a potentially suicidal patient. In nonclinical settings, publicly available, user-generated text may be a less expensive and less invasive data collection method for identifying persons at risk of suicide or monitoring trends in suicidal ideation. For people identified as at risk for suicide who are in nonclinical settings, resources such as crisis text lines could be made available instantly and electronically.

In addition to providing a potentially lower-cost, and faster, alternative to traditional assessments of suicidal risk, NLP-based modeling has the potential to reduce existing disparities. Groups such as racial/ethnic minorities, young adults, and individuals from lower socioeconomic strata may often be absent from traditional suicidal risk monitoring datasets because they may face access barriers to health services generally. Lacking health system surveillance of these individuals, NLP has the potential to be applied more broadly to capture suicide risk and direct resources through low-cost interventions such as the text-message system used in this analysis, which could be deployed within vulnerable communities. These methods may also be useful for capturing emerging psychiatric issues among high-risk youth, possibly assisting in communications between schools, families, youth services, and law enforcement to ensure that these children receive proper treatment before detrimental academic, social, and criminal justice outcomes.

## 5. Conclusion

It is possible to use natural language processing- (NLP-) based machine learning prediction methods to predict suicide risk as well as heightened psychiatric symptoms in free-text responses sent via mobile phone. In the global effort to prevent the staggering emotional, social, and economic impact of suicide, the use of novel NLP methods may be used to create low-cost and effective alternatives to traditional resource-heavy data monitoring systems. Ultimately, applying NLP technology for this purpose may contribute significantly to identifying, and then intervening with, persons who are at heightened risk of suicide. This will contribute to the ultimate goal of reducing suicides and suicide attempts, even in low-resource settings.

## Figures and Tables

**Table 1:** Baseline characteristics by suicidal ideation status and average GHQ-12 ≥ 4 (*n* = 1,453).

Mean (SD) or %	Suicidality			GHQ-12		
	Never suicidal	Ever suicidal	*p* value	Avg GHQ < 4	Avg GHQ ≥ 4	*p* value
	*n* = 609	*n* = 844		*n* = 662	*n* = 796	
Age (cont)	40.0 (13.8)	41.6 (13.9)	<0.001	40.9 (0.5)	40.2 (0.5)	0.316
Percent female	59.3	69.3	<0.001	59.7	69.7	<0.001
Average nightly sleep (hours)	7.3 (1.6)	6.9 (1.9)	<0.001	7.4 (1.4)	6.8 (2.0)	<0.001
Self-rated sleep quality (0–100)	72.4 (24.1)	55.0 (25.1)	<0.001	73.6 (21.2)	52.8 (26.2)	<0.001
Self-rated anger rarely (0–100)	83.8 (16.3)	67.0 (23.5)	<0.001	83.1 (15.6)	66.6 (24.3)	<0.001
Self-rated changes in appetite (0–100)	55.0 (18.8)	50.0 (23.0)	<0.001	56.2 (17.8)	48.7 (23.5)	<0.001
Medication adherence (0–100)	76.5 (36.7)	75.2 (35.1)	0.005	77.1 (35.4)	74.5 (36.0)	0.012
Average WHO-5	61.6 (21.1)	38.3 (20.0)	<0.001	63.3 (18.9)	35.0 (18.6)	<0.001

Significance was assessed using 2-sample *t*-tests for continuous normal variables, Wilcoxon rank-sum (Mann-Whitney) tests for continuous skewed variables, and Pearson chi-square test for binary variables.

**Table 2:** Structured variable predictors of suicidal ideation and average GHQ-12 ≥ 4 (*n* = 1,453).

	Suicidal ideation ever (OR, 95% CI)	*p* value	GHQ ≥ 4 (OR, 95% Cl)	*p* value
Age (cont)	1.02 (1.01–1.03)	<0.001	0.99 (0.98–1.00)	0.2
Female	1.12 (0.84–1.49)	0.456	1.02 (0.74–1.40)	0.913
Average nightly sleep (hours)	1.11 (1.01–1.22)	0.037	0.98 (0.88–1.09)	0.735
Sleep quality (0–100)	0.99 (0.99–1.00)	0.094	0.99 (0.98–1.00)	0.029
Anger rarely (0–100)	0.97 (0.96–0.98)	<0.001	0.98 (0.97–0.99)	<0.001
Changes in appetite (0–100)	1.00 (1.00–1.00)	0.482	1.00 (0.99–1.00)	0.793
Medication adherence (0–100)	1.00 (1.00–1.00)	0.746	1.00 (0.96–1.00)	0.912
WHO_5 wellbeing scale	0.96 (0.95–0.97)	<0.001	0.94 (0.93–0.95)	<0.001

Both models were completed using logistic regression (STATA 14 software).

**(a):** 

Probability	Spanish	English
0.35 to <0.4	conté	I told

0.3 to <0.35	monotona, Equasim (Ritalin), acosado, trabajamos	Monotony, Ritalin, harassed, we work

0.25 to <0.3	raza, aseos, resfriado, pronuncio	Race, restrooms, congested (sick), I pronounce

0.2 to <0.25	rechaza, jornada, será, suave, endoscopia, ello, ganas, solo, dedicados, recurrente, morirá, probado, causa, inevitable, raíz, digo	Rejects, work shift, will be, soft, endoscopy, it, desire, alone, dedicated, recurrent, will die, tried, cause, inevitable, root, I say

0.15 to <0.20	lloré, primero, traumatólogo, hilos, alpina, reumatóloga, brazo, rigidez, estallar, cumplen, desapareciendo, agobia, vulnerable, síntomas, ganar, preferiría, culpas, explico, recordando, teoría, adelgazara, gasolina, emocionalmente	I cried, first, trauma surgeon, threads, alpine, rheumatologist, arm, stiffness, explode, they obey, disappearing, overwhelms, vulnerable, symptoms, win, prefer, blame, I explain, remembering, theory, will lose weight, gasoline, emotionally

**(b):** 

Probability	Spanish	English
0.35 to <0.45	recorrer, horarios, comunicar	Wander, schedules, communicate

0.3 to <0.35	iré, parecida, gimnasia, destacando, ido	Will go, similar, gym, highlight, going

0.25 to <0.3	fumada, llevaré, desactivado, crema, gris	Smoked, will bring, disarmed, cream, grey

0.2 to <0.25	hipnotizada, funcionar, manual, futuro, mala, deprimida, recogas, me voy, tardado, esclavo, síntomas, golpe, lado, merienda, primera, inspectora, valoren, suave, entendido	Hypnotized, to function, manual, future, bad, depressed, pick, I'm leaving, I took, slave, symptoms, hit, side, snack, first, inspector, worth, soft, understood

0.15 to <0.20	Cerca, subdirectora, porta, Francia, cumplido, suicidios, bastan, videojuegos, laxante, con, voluntariado, particularmente, empleados, cabeza, contesto, confiaba, realizado	Near, subdirector, port, France, completed, suicides, suffice, video games, laxative, with, volunteered, particularly, employees, head, answer, trust, realized

**Table 4:** Distribution of suicidal ideation status by GHQ-12 cutoff ≥ 4.

Suicidal ideation ever	Avg GHQ-12 < 4	Avg GHQ-12 ≥ 4	Total	*p* value
No (*n*, row%)	433 (70.9%)	178 (29.1%)	611	*p* < 0.001
Yes (*n*, row%)	229 (27.0%)	618 (73.0%)	847	

Total	662 (45.4%)	796 (54.6%)	1,458	

## Appendix

See Table 4.
